# Squeezed vacuum interaction with an optomechanical cavity containing a quantum well

**DOI:** 10.1038/s41598-022-07436-5

**Published:** 2022-03-07

**Authors:** H. Jabri, H. Eleuch

**Affiliations:** 1 Higher Institute of Biotechnology of Beja, University of Jendouba, Beja, 9000 Tunisia; 2 Department of Applied Physics and Astronomy, University of Sharjah, Sharjah, 27272 United Arab Emirates; 3 Department of Applied Sciences and Mathematics, College of Arts and Sciences, Abu Dhabi University, Abu Dhabi, 59911 United Arab Emirates; 4 Institute for Quantum Science and Engineering, Texas A&M University, College Station, TX 77843 USA

**Keywords:** Physics, Optical physics, Quantum optics

## Abstract

We investigate a hybrid system consisting of an optomechanical resonator and an optical cavity containing a quantum well. The system is coupled to a squeezed vacuum reservoir. We analyze the effect of the injection of squeezed photons inside the cavity on the intensity spectrum. The system reaches a regime of hybrid resonance where mechanical, excitonic and cavity modes are intermixed. Despite that the optomechanical interaction is the source of the nonlinearity in the system, the optimum squeezing is obtained at the hybrid resonance frequencies. However, when the squeezed vacuum is applied, at these frequencies the minimum squeezing is realized as well as an increase of fluctuations is observed. We show that the squeezed vacuum transforms the coherent states into highly squeezed states of light, and offers a great flexibility to obtain maximal squeezing. Furthermore, a perfect squeezing is predicted.

## Introduction

During the last decade, considerable efforts have been dedicated to optomechanical systems. These investigations explore the nonlinear interaction between an optical cavity and a mechanical oscillator through a radiation pressure force^1–6^. Such systems have several applications including creation of mechanical motion and nonclassical states of light, highly sensitive measurement and quantum information^1,3,7–9^. Interest, of both applied and fundamental aspects, is also justified by numerous proposals and experimental observations that includes the optical cooling of a resonator to its quantum mechanical ground state^10^ and observation of its zero-point motion^11^, photon blockade and single-photon emission^12,13^, optomechanical entanglement^14,15^, the observation of back-action effects^16^, optical bistability^17^, optomechanically induced transparency^18,19^, detection of possible quantum gravitation effects^20^, and many others.

Among these interests, generation of squeezed states of light using optomechanical devices is still one of the most interesting fields of quantum optics over the recent years^1,2,21^. This property of light is an important resource for many applications, such as in ultra-sensitive measurements^19,20^, quantum cryptography^24–26^, gravitational wave detection^27–30^, quantum computing^31–36^, sub-shot-noise interferometry^37–39^ and quantum limited displacement sensing^40^. Generally, nonlinear interactions in a quantum system are responsible for the appearance of squeezed states. Excitonic nonlinearity in quantum wells is an illustrative example^41–45^. In this perspective, via a new emerging setup namely the dipolariton cavity, we can achieve a high amount of squeezing with an excellent resistance to thermal excitations. This system combines two interacting quantum wells, where additional nonlinearities enhance the degree of squeezing^46–49^.

In this paper, we explore the correlations of photons and the quantum statistics of an optomechanical cavity containing a quantum well and coupled to a squeezed vacuum reservoir. By determining intensity and squeezing spectra, we analyze the properties of light produced by the system. The effect of the quantum well and the squeezed vacuum are discussed in details. We show that the presence of excitons tends to break the bistable behavior. Our study reveals the existence of hybrid resonances appearing in the system, a mixture of states of excitonic, mechanical and cavity modes. Moreover, in this hybrid system, the squeezed vacuum greatly affects the intensity spectrum especially at the hybrid resonant regime. More interestingly, we show that the system produces a good amount of squeezing due to the nonlinear optomechanical coupling. This squeezing can be strongly enhanced and rigorously stabilized by applying the squeezed vacuum. The studied scheme would give more possibilities and alternatives in the field of the squeezing generators.

The paper is organized as follows. In Sect. “Model Hamiltonian and dynamics”, the system’s Hamiltonian is presented and the time evolution equations are derived. In the third section, we determine the steady-state solutions and discuss the photonic intensity and the optical bistability. Section “Fluctuation dynamics” is devoted to the quantum fluctuation analysis of the cavity field. The intensity power spectrum is studied in Sect. “Intensity spectrum and hybrid resonances”. In the last section, we focus on the squeezing of light as a function of the frequency, the frequency detuning, the squeeze parameter and the optomechanical coupling.

## Model Hamiltonian and dynamics

**Figure 1:**
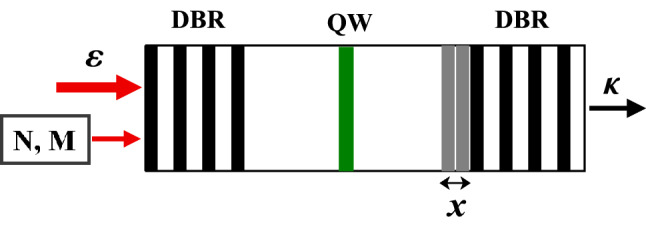
Scheme of the system. A cavity formed by two distributed Bragg reflectors (DBR), in which is placed a quantum well (QW). The mirror is subject of a mechanical motion *x*. GaAs (black stripes) and AlAs (white stripes) layers are used. The cavity is coherently driven by an external laser of amplitude $\varepsilon$ and coupled to a squeezed reservoir with parameters *N* and *M*. The injected squeezed light interacts with the quantum well and affects the mechanical resonator. $\kappa$ represents the damping rate of the cavity photon.

We consider a mechanical resonator with moveable Bragg reflectors and a quantum well embedded in a single-mode cavity as shown in the schematic representation of Fig. 1. The cavity is coherently pumped by a field of frequency $\omega _{p}$. The mechanical resonator undergoes a force related to the mean number of photons inside the cavity. By using a high quality factor mirror, it is possible to attain the regime of strong coupling between cavity photons and excitons. The study is restricted to a single mechanical mode $\omega _{m}$. It is obtained when the detection bandwidth includes only one single mechanical resonance and a negligible mode-mode coupling^50^. This is justified by the adiabatic limit in which the frequency of the moveable mirror is much smaller than the free spectral range of the cavity^49^. We should note that in this limit, the photon number produced by the Doppler, Casimir and retardation effects is negligible^52,53^.

We neglect the spin degrees of freedom. In the plane of the semiconductor layers, we have a translational invariance. This property means that excitons having a wavevector $K_{\parallel }$ is only coupled with radiation possessing an equal wavevector $k_{\parallel }$. Additionally, the exciton and cavity modes are quantized in the direction normal to the layers. Then, a strong coupling can take place by considering the interaction between an excitonic mode and a photonic mode only. We also consider that the modes of interest are weakly coupled to the other exciton modes form a thermal reservoir^43^.

Additionally, we consider that a squeezed vacuum reservoir, with a squeeze parameter *r*, acts on the cavity. The assumption of the single cavity-mode is justified by the adiabatic limit, i.e., $\omega _{m} \ll \pi c/L$ where *L* represents the cavity length in the absence of the cavity field and *c* denotes the speed of light in vacuum^50^.

The total Hamiltonian of the system, in a frame rotating at the drive frequency $\omega _{p}$, is given by:

1 $$\begin{aligned} H =&-\Delta _{a}a^{\dagger }a-\Delta _{b}b^{\dagger }b+\omega _{m}c^{\dagger }c +i\varepsilon ( a^{\dagger }-a)\nonumber \\&+ig( a^{\dagger }b-b^{\dagger }a)-g_{0}a^{\dagger }a(c+c^{\dagger }) , \end{aligned}$$

where $a^{\dagger }$ (*a*), $b^{\dagger }$ (*b*) and $c^{\dagger }$ (*c*) represent the creation (annihilation) operators of cavity, excitonic and mechanical modes, respectively. The parameter *g* characterizes the strength of the photon-exciton coupling. $g_{0}$ is the single-mode optomechanical coupling rate of the optomechanical interaction. The amplitude of the drive laser is given by $\varepsilon =\sqrt{\kappa P/\hbar \omega _{p}}$, where $\kappa$ and *P* are the cavity damping rate and the laser power, respectively. $\Delta _{a}=\omega _{p}-\omega _{c}$ and $\Delta _{b}=\omega _{p}-\omega _{ex}$ represent the frequency detunings between laser pump, exciton and cavity modes. The evolution equations of the three modes of our hybrid system are written as:

2 $$\begin{aligned} {\dot{a}}&=\left( -\frac{\kappa }{2}+i\Delta _{a}\right) a+gb+ig_{0}a(c+c^{\dagger })+\varepsilon +\sqrt{\kappa }a_{in}, \end{aligned}$$

3 $$\begin{aligned} {\dot{b}}&=\left( -\frac{\gamma }{2}+i\Delta _{b}\right) b-ga+\sqrt{\gamma }b_{in}, \end{aligned}$$

4 $$\begin{aligned} {\dot{c}}&=-\left( \frac{\gamma _{m}}{2}+i\omega _{m}\right) c+ig_{0}a^{\dagger }a+\sqrt{\gamma _{m}}c_{in}, \end{aligned}$$

where $\gamma _{m}$ is the decay rate of the mechanical oscillator, and $\gamma$ represents the spontaneous emission rate of excitons. $a_{in}$, $b_{in}$, and $c_{in}$ denote the Langevin noise operators for cavity, exciton, and mechanical modes, respectively.

## Steady-state solutions and stability analysis

**Figure 2:**
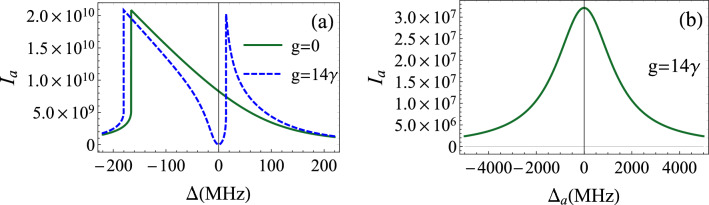
Cavity photonic intensity $I_{a}$ as a function of the detunings for $\gamma _{m}=40$ Hz, $\kappa =2\pi \times 10^{5}$ Hz, $g_{0}=300$ Hz, $\omega _{m}=2\pi \times 2.7$ MHz, $\varepsilon =12.74\times 10^{6}\kappa$ and $\gamma =3.6$ MHz. (**a**) Equal detunings $\Delta _{a}=\Delta _{b}=\Delta$. (**b**) Photon-exciton resonance $\Delta _{b}=0$.

The evolution of the mean fields can be derived from the set (2)-(4), then the steady-state solutions for mechanical and excitonic modes are written as:

5 $$\begin{aligned} \langle c\rangle =\frac{ig_{0}I_{a}}{\frac{\gamma _{m}}{2}+i\omega _{m}} ; \; \langle b\rangle =\frac{g}{-\frac{\gamma }{2}+i\Delta _{b}}\langle a\rangle , \end{aligned}$$

while the cavity mean-field is given by:

6 $$\begin{aligned} \langle a\rangle =\frac{\varepsilon }{\kappa _{0}+i(\Delta _{a0}-\alpha _{m}I_{a})}, \end{aligned}$$

where $I_{a}=\langle a^{\dagger }\rangle \langle a\rangle$ is the photonic intensity, and the other terms are defined by:

7 $$\begin{aligned} \kappa _{0}=\frac{\kappa }{2}+\frac{\frac{\gamma }{2} g^{2}}{(\frac{\gamma }{2})^{2}+\Delta _{b}^{2}} ; \; \Delta _{a0}=-\Delta _{a}+\frac{g^{2}\Delta _{b}}{(\frac{\gamma }{2})^{2}+\Delta _{b}^{2}} ; \;\alpha _{m}&=\frac{2g_{0}^{2}\omega _{m}}{(\frac{\gamma _{m}}{2})^{2}+\omega _{m}^{2}}. \end{aligned}$$

From this, we can deduce a simple relation linking photonic, excitonic and mechanical modes intensities as:

8 $$\begin{aligned} \frac{I_{c}}{I_{b}}=\frac{\beta _{m}}{\beta _{b}}I_{a}, \end{aligned}$$

where $\beta _{b}=g^{2}/((\frac{\gamma }{2})^{2}+\Delta _{b}^{2})$ and $\beta _{m}=g_{0}^{2}/((\frac{\gamma _{m}}{2})^{2}+\omega _{m}^{2})$. From Eq. (6), we deduce the following cubic equation in $I_{a}$:

9 $$\begin{aligned} I_{a}\left[ \kappa _{0}^{2}+(\Delta _{a0}-\alpha _{m}I_{a})^{2}\right] =|\varepsilon |^{2}. \end{aligned}$$

**Figure 3:**
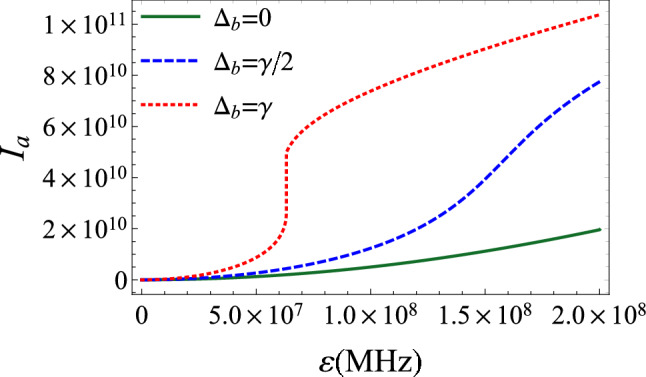
Cavity photonic intensity $I_{a}$ as a function of the amplitude of the coherent drive $\varepsilon$ for $\gamma _{m}=40$ Hz, $\kappa =2\pi \times 10^{5}$ Hz, $g_{0}=300$ Hz, $\Delta =\omega _{m}=2\pi \times 2.7$ MHz, $\gamma =3.6$ MHz and $g=14\gamma$. The regime of the optical bistability appears from a certain value of the photon-exciton detuning $\Delta _{b}$ (dotted line). Before this threshold, the bistability disappears and the system is fully stable for $\Delta _{b}=0$ (solid line).

To study the bistability property, we should calculate $\partial |\varepsilon |^{2}/\partial I_{a}=0$. From which we deduce the bistability condition for our system as $\Delta _{a0}^{2}-3\kappa _{0}^{2}>0$. Using the terms defined in Eq. (7), this condition becomes:

10 $$\begin{aligned} \left[ \Delta _{a}^{2}-3\left( \frac{\kappa }{2}\right) ^{2} \right] -2\beta _{b}\left( \Delta _{a} \Delta _{b}+3\frac{\kappa }{2}\frac{\gamma }{2}\right) +\beta _{b}^{2}\left[ \Delta _{b}^{2}-3\left( \frac{\gamma }{2}\right) ^{2} \right] >0. \end{aligned}$$

Even though the nonlinear optomechanical interaction is responsible of the bistable behavior, the previous equation does not contain any information concerning the mechanical part of the system. The bistability is governed by photonic and excitonic parameters. In the absence of the quantum well ($\beta _{b}=0$), Eq. (10) reduces simply to the condition for the optomechanical cavity, $\Delta _{a}^{2}-3(\frac{\kappa }{2})^{2}>0$.

In all plots of this work, we choose physical parameters that are experimentally realizable and used in optomechanical systems possessing high quality factor $Q_{m} = \omega _{m}/\gamma _{m} \approx 10^{6}$^54^. Furthermore, we suppose that the mechanical mode frequency $\omega _{m}$ is greater than the cavity damping rate $\kappa$. This corresponds to the good cavity limit, a prerequisite for resolved sideband cooling of micromechanical resonators^55^.

The photonic intensity against the frequency detuning is plotted in Fig. 2. In Fig. 2a we assume that $\Delta _{a}=\Delta _{b}=\Delta$. The solid line representing the photonic intensity in the absence of the quantum well indicates that $I_{a}$ shows a signature of the optical bistability. When excitons come into play, a second peak appears in the positive detuning region, relative to polaritons (dashed line). Considering a resonant interaction between the excitonic mode and the coherent drive ($\Delta _{b}=0$), the bistable behaviour disappears. In this case, the intensity is formed by a single peak around total resonance (Fig. 2b). This is interesting due to the fact that the bistability depends on the value of $\Delta _{b}$. To focus on this aspect, we plot in Fig. 3 the mean intracavity photon number against the amplitude of the coherent drive. It is clearly shown that the bistability appears for a certain value of $\Delta _{b}$. Before this limit, the system is stable essentially near zero pump-exciton detuning. This can be explained by the contribution of the last term in Eq. (10). If it is less than zero, $\Delta _{b}^{2}<3(\frac{\gamma }{2})^{2}$, the bistable behavior is reduced or even disappears. The presence of excitons in the cavity tends to vanish this bistability. We also note that the inequality $\Delta _{b}^{2}>3(\frac{\gamma }{2})^{2}$ is nothing else than the bistability condition for semiconductor microcavity with a quantum well^56^. The effect of the excitonic mode on the bistability behavior can be explained as follows; the quantum well is an additional degree of freedom for the system. A part of the injected photons are involved in the exciton-photon interaction as the coherent pump increases (especially for resonant excitation, $\Delta _{b}=0$). For this, $I_{a}$ varies monotonously and no appearance of the hysteresis. This means that the quantum well needs more pumped light that is consumed in the light-matter interaction. Based on this assertion, in the following we work with the assumption $\Delta _{b}=0$, where the system is fully stable.

## Fluctuation dynamics

To linearize the quantum Langevin equations, each field operator can be presented as the sum of a mean-field value and a fluctuation part. Then, we have $a=\langle a\rangle +\delta a$, $b=\langle b\rangle +\delta b$ and $c=\langle c\rangle +\delta c$. From there, the evolution of the fluctuations is given by:

11 $$\begin{aligned} \delta {\dot{a}}&=\left( -\frac{\kappa }{2}+i(\Delta _{a}-\alpha _{m}I_{a})\right) \delta a+g \delta b+G_{0}(\delta c+\delta c^{\dagger }) +\sqrt{\kappa }a_{in}, \end{aligned}$$

12 $$\begin{aligned} \delta {\dot{b}}&=\left( -\frac{\gamma }{2}+i\Delta _{b}\right) \delta b-g\delta a+\sqrt{\gamma }b_{in}, \end{aligned}$$

13 $$\begin{aligned} \delta {\dot{c}}&=\left( -\frac{\gamma _{m}}{2}+i\omega _{m}\right) \delta c+G_{0}(\delta a^{\dagger }-\delta a)+\sqrt{\gamma _{m}}c_{in}, \end{aligned}$$

where $G_{0}=g_{0}\sqrt{\langle a\rangle \langle a^{\dagger }\rangle }=g_{0}\sqrt{I_{a}}$ represents the optomechanical coupling strength. For simplicity, the phase of the driving laser has been chosen such that $\langle a\rangle =-i|\langle a\rangle |$. When the system interacts with a squeezed vacuum, the noise operator of the cavity field $a_{in}$, appearing in Eq. (11), satisfies:

14 $$\begin{aligned} \langle a_{in}(t) a_{in}^{\dagger }(t') \rangle&=(N+1)\delta (t-t'), \end{aligned}$$

15 $$\begin{aligned} \langle a^{\dagger }_{in}(t) a_{in}(t') \rangle&=N \delta (t-t'), \end{aligned}$$

16 $$\begin{aligned} \langle a_{in}(t) a_{in}(t') \rangle&=\langle a^{\dagger }_{in}(t) a^{\dagger }_{in}(t') \rangle =M \delta (t-t'), \end{aligned}$$

where $M=e^{i\varphi } \text {sinh} (r) \text {cosh} (r)$ and $N=\text {sinh}^{2}(r)$. *M* is the two-photon correlation of the squeezed reservoir and *N* denotes the mean photon number. *r* is the squeezing parameter and $\varphi$ being the phase of the squeezed radiation. Then, the parameters *M* and *N* can be linked simply by $M=\sqrt{N(N+1)}e^{-i\varphi }$^55^. For simplicity, we consider that $\varphi =0$. Furthermore, the only non-zero fluctuation correlations relative to excitonic noise is given by $\langle b_{in}(t) b_{in}^{\dagger }(t') \rangle =\delta (t-t')$. The mechanical mode is affected by a viscous force with a damping rate $\gamma _{m}$ and also by a Brownian stochastic force having a zero mean value $\xi$ and obeys the following correlation function^51,58–60^:

17 $$\begin{aligned} \langle \xi (t) \xi (t') \rangle =\frac{\gamma _{m}}{\omega _{m}} \int \frac{d\omega }{2\pi } e^{-i\omega (t-t')} \omega \left[ \text {coth}\left( \frac{\hbar \omega }{2k_{B}T}\right) +1\right] , \end{aligned}$$

where $k_{B}$ denotes the Boltzmann constant and *T* represents the temperature of the reservoir of the mechanical oscillator. This Brownian noise $\xi (t)$ is, in general, a non-Markovian Gaussian noise, but in the limit of high-temperature mechanical reservoir, it is possible to make the following Markovian approximation to the quantum Brownian noise $\xi (t)$:

18 $$\begin{aligned} \langle \xi (t) \xi (t') \rangle \simeq \gamma _{m} (2n_{th}+1) \delta (t-t'), \end{aligned}$$

where $n_{th}=\{\text {exp}[\hbar \omega _{m}/(k_{B}T)]-1\}^{-1}$ represents the thermal phonon number of the mechanical oscillator. Then, via suitable transformations and in the limit of large $\omega _{m}$^61^, the mechanical noise operators satisfy to:

19 $$\begin{aligned} \langle c_{in}(t) c^{\dagger }_{in}(t') \rangle&=(n_{th}+1) \delta (t-t'), \end{aligned}$$

20 $$\begin{aligned} \langle c^{\dagger }_{in}(t) c_{in}(t') \rangle&=n_{th} \delta (t-t'). \end{aligned}$$

Generally, it is more appropriate to work in frequency domain. For this, the set of Eqs. (11)–(13) can be rewritten in Fourier space, in matrix form as $M(\omega )H(\omega )=K(\omega )$, where $H(\omega )=(\delta a,\delta b, \delta c, \delta a^{\dagger },\delta b^{\dagger },\delta c^{\dagger })^{T}$, $K(\omega )$=($\sqrt{\kappa } a_{in}$,$\sqrt{\gamma } b_{in}$, $\sqrt{\gamma _{m}} c_{in}$,$\sqrt{\kappa } a_{in}^{\dagger }$, $\sqrt{\gamma } b_{in}^{\dagger }$,$\sqrt{\gamma _{m}} c_{in}^{\dagger }$)$^{T}$ and

21 $$\begin{aligned} M(\omega )=\left( \begin{array}{cccccc} \mu _- & -g & -G_0 & 0 & 0 & -G_0 \\ g & \nu _- & 0 & 0 & 0 & 0 \\ G_0 & 0 & \xi _- & -G_0 & 0 & 0 \\ 0 & 0 & -G_0 & \mu _+ & -g & -G_0 \\ 0 & 0 & 0 & g & \nu _+ & 0 \\ -G_0 & 0 & 0 & G_0 & 0 & \xi _+ \\ \end{array} \right) , \end{aligned}$$

where $\mu _{\mp }=\frac{\kappa }{2}+i \left( \omega \mp \left( \Delta _a- \alpha _m I_{a}\right) \right)$, $\nu _{\mp }=\frac{\gamma }{2}+i \left( \omega \mp \Delta _b\right)$ and $\xi _{\mp }=\frac{\gamma _m}{2}+i \left( \omega \mp \omega _m\right)$. Solving such a matrix equation for the fluctuation operator of the cavity field $\delta a$ yields:

22 $$\begin{aligned} \delta a(\omega )=&\sqrt{\kappa } \alpha _{1} a_{in}+\sqrt{\gamma } \alpha _{2} b_{in}+\sqrt{\gamma _{m}}\alpha _{3} c_{in} \nonumber \\&+ \sqrt{\kappa }\alpha _{4} a_{in}^{\dagger }+\sqrt{\gamma }\alpha _{5} b_{in}^{\dagger }+\sqrt{\gamma _{m}}\alpha _{6} c_{in}^{\dagger }, \end{aligned}$$

where the coefficients $\alpha _{i}(\omega )$ are defined by:

23 $$\begin{aligned} \alpha _{1} =&\frac{\nu _{-}}{d} \left[ \xi _- \xi _+ \left( g^2+\mu _+ \nu _+\right) +G_0^2 \nu _+ \left( \xi _--\xi _+\right) \right] , \end{aligned}$$

24 $$\begin{aligned} \alpha _{2}=&\frac{g}{d} [\xi _- \xi _+ \left( g^2+\mu _+ \nu _+\right) + G_0^2 \nu _+ \left( \xi _--\xi _+\right) ], \end{aligned}$$

25 $$\begin{aligned} \alpha _{3}=&\frac{G_{0}}{d} \nu _- \xi _+ \left( g^2+\mu _+ \nu _+\right) ;\quad \alpha _{4}=\frac{G_{0}^{2}}{d} \nu _- \nu _+ \left( \xi _+-\xi _-\right) , \end{aligned}$$

26 $$\begin{aligned} \alpha _{5}=&\frac{g}{d} G_0^2 \nu _- \left( \xi _+-\xi _-\right) ;\quad \alpha _{6}=\frac{G_{0}}{d} \nu _- \xi _- \left( g^2+\mu _+ \nu _+\right) , \end{aligned}$$

and

27 $$\begin{aligned} d=&\,\xi _- \xi _+ (g^2+\mu _- \nu _-) (g^2+\mu _+ \nu _+)-G_0^2 (\xi _--\xi _+) \nonumber \\&\times [\nu _- (g^2+(\mu _+-\mu _-) \nu _+)-g^2 \nu _+]. \end{aligned}$$

## Intensity spectrum and hybrid resonances

In order to examine the intensity power spectrum of the cavity field, we need to calculate the Fourier transform of the two-time correlation $\langle \delta a^{\dagger }(t+\tau ) \delta a (t) \rangle$:

28 $$\begin{aligned} S_{inside}(\omega )=\int _{-\infty }^{+\infty }d\tau \langle \delta a^{\dagger }(t+\tau ) \delta a (t) \rangle e^{-i(\omega -\omega _{0})\tau } =C_{a^{\dagger }a}(\omega ). \end{aligned}$$

The term appearing on the right side of Eq. (28) is given by $2\pi C_{a^{\dagger }a}(\omega ) \delta (\omega +\omega ')=\langle \delta a^{\dagger }(\omega ) \delta a (\omega ') \rangle$. Then, with the help of Eq. (22), we get:

29 $$\begin{aligned} C_{a^{\dagger }a}( \omega ) =&\kappa M \alpha _{1}^{*}( \omega ) \alpha _{4}( -\omega )+ \kappa N| \alpha _{1}( -\omega ) | ^{2} \nonumber \\&+\kappa M \alpha _{4}^{*}( \omega ) \alpha _{1}( -\omega ) +\kappa ( N+1) | \alpha _{4}( -\omega ) | ^{2} \nonumber \\&+\gamma | \alpha _{5}( -\omega ) | ^{2}+\gamma _{m} n_{th}| \alpha _{3}( -\omega ) | ^{2} \nonumber \\&+\gamma _{m} (n_{th}+1) | \alpha _{6}( -\omega ) | ^{2}. \end{aligned}$$

Light outgoing the cavity is received by photodetectors, then analyzed. Thus, it is more appropriate to determine the intensity spectrum outside the cavity $S(\omega )$. Indeed, the standard input-output relation $\delta a^{out}=\sqrt{\kappa }\delta a-a^{in}$^62^, allows us to obtain $S(\omega )=C_{a^{\dagger }a}^{out}( \omega ) = \kappa C_{a^{\dagger }a}(\omega ) -2\kappa N \text {Re}( \alpha _{1}( -\omega ) ) -2\kappa M \text {Re}( \alpha _{4}( -\omega ) )+ N$.

**Figure 4:**
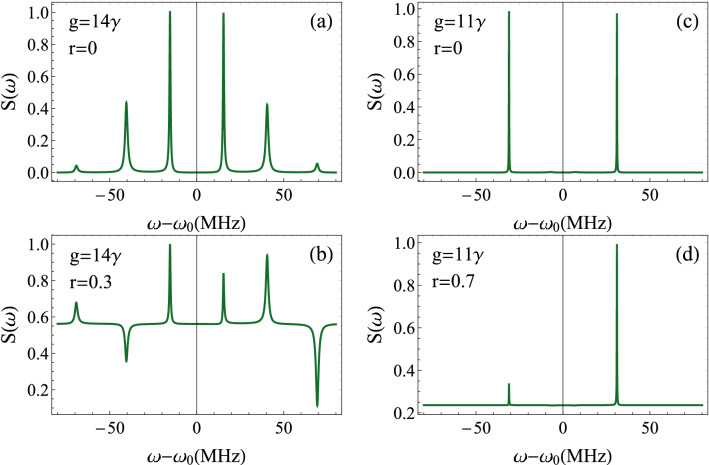
Intensity spectrum of the transmitted field $S(\omega )$ as a function of the frequency $\omega -\omega _{0}$ for $\gamma =3.6$ MHz, $\gamma _{m}=40$ Hz, $\kappa =2\pi \times 10^{5}$ Hz, $g_{0}=300$ Hz, $n_{th}\simeq 833$ corresponding to a reservoir temperature $T=0.4$ K, $\Delta =\omega _{m}=2\pi \times 2.7$ MHz, $\varepsilon =1.6\times 10^{8}\kappa$ and $\Delta _{b}=0$. (**a**) and (**b**) The peaks correspond to mechanical, excitonic and optical modes. (**c**) and (**d**) The system approaches the hybrid resonance regime. In both cases, the squeezed vacuum affects the intensity peaks and provokes a dissymmetry in the spectrum.

Before studying the effect of the squeezed vacuum, we should first understand the intensity spectrum in the absence of external squeezed photons. The number and nature of peaks of the spectrum can be explained based on of the eigenvalues of the system provided by Eqs. (11)–(13). These eigenvalues can be written as:

30 $$\begin{aligned} \lambda _{1,2}&=\frac{1}{4}(\gamma +\kappa )+\frac{i}{4} \left( 2 {\tilde{\Delta }} \pm \sqrt{16 g^2+(2 {\tilde{\Delta }} -i (\gamma -\kappa ))^2} \right) , \end{aligned}$$

31 $$\begin{aligned} \lambda _{3,4}&=\frac{1}{4}(\gamma +\kappa )-\frac{i}{4} \left( 2 {\tilde{\Delta }} \pm \sqrt{16 g^2+(2 {\tilde{\Delta }} -i (\gamma -\kappa ))^2} \right) , \end{aligned}$$

32 $$\begin{aligned} \lambda _{5,6}&=\frac{1}{2} (-\gamma _m\pm 2 i \omega _m), \end{aligned}$$

where ${\tilde{\Delta }}$ is the effective detuning given by ${\tilde{\Delta }}=\Delta _{a}-\alpha _{m}I_{a}$. These six eigenvalues correspond to six peaks in the spectrum. As we can see, the six peaks appear on the intensity spectrum of Fig. 4 for $g=14\gamma$. Now, we have to distinguish the optomechanical and polaritonic resonances. The peaks centered around $\omega -\omega _{0}\approx \pm \omega _{m}\approx \pm 16.95$MHz, are for the optomechanical resonances. However, according to Eqs. (30) and (31), and for large coupling *g*, we should observe two peaks around $\omega -\omega _{0}\approx \pm ({\tilde{\Delta }}+\sqrt{{\tilde{\Delta }}^{2}+4g^{2}})/2\approx g$, signature of the photon-exciton coupling (polaritons). By reducing the coupling, ${\tilde{\Delta }}$ becomes larger and these last peaks are split, leading to four hybrid peaks of mechanical, excitonic and cavity modes (Fig. 4a). Two of the hybrid peaks are centered around $\omega -\omega _{0}\approx \pm ({\tilde{\Delta }}-\sqrt{{\tilde{\Delta }}^{2}+4g^{2}})/2\approx 41$MHz. By applying the external squeezed field ($r=0.3$), the whole system gains in photonic intensity. The optomechanical resonances are no more dominant in the spectrum, with an increasing relative intensity of two of the hybrid peaks. In counterpart, the two other hybrid peaks correspond to minimal intensity (Fig. 4b). Such a behavior can be explained as follows. As the number of photons increases in the cavity due to the squeezed injection, this generates considerable photon pressure on the resonator. In turn, this enhances the amplitude of the mechanical mode and leads to optomechanical resonance, at the expense of decreased amplitudes of a part of the hybrid resonances.

For $g=11\gamma$, the optomechanical resonances are highly reduced and the hybrid peaks of the previous situation completely vanish. We observe two dominant peaks in the spectrum (Fig. 4c). This picture corresponds to the first stage before the total disappearance of the pure polaritonic and optomechanical resonances, and the emergence of the hybrid resonance regime. Keeping the same parameters values as in Fig. 4c and increasing *r* to 0.7, we observe that the intensity of the negative relative-frequency peak decreases. The other resonant peak is unaffected, and the spectrum is asymmetric (Fig. 4d).

## Squeezing spectrum: injected vs intracavity squeezing

Here, we explore the squeezing of the transmitted radiation due to the optomechanical coupling and the effect of the injected squeezed photons on the dynamical behavior. The squeezing spectrum of the field outside the cavity is defined as^63^:

33 $$\begin{aligned} S_{\theta }(\omega )&=\int _{-\infty }^{+\infty } \langle \delta X_{\theta }^{out}(t+\tau ) \delta X_{\theta }^{out}(t) \rangle _{ss} e^{-i(\omega -\omega _{0}) \tau } d\tau \nonumber \\&=\langle \delta X_{\theta }^{out}(\omega ) \delta X_{\theta }^{out}(\omega ) \rangle , \end{aligned}$$

where $X_{\theta }^{out}(\omega ) = e^{-i\theta } \delta a^{out}(\omega )+e^{i\theta } \delta a^{\dagger out}(\omega )$ is a quadrature of the cavity field, whereas $\theta$ designs its controllable phase. Indeed, Eq. (33) becomes $S_{\theta }(\omega )=C_{aa}^{out}(\omega ) e^{-2i\theta }+C_{a^{\dagger }a^{\dagger }}^{out}(\omega ) e^{2i\theta }+C_{aa^{\dagger }}^{out}(\omega )+C_{a^{\dagger }a}^{out}(\omega )$, where $C_{aa}^{out}(\omega )$ is given by, $2\pi C_{aa}^{out}(\omega ) \delta (\omega +\omega ')=\langle \delta a^{out}(\omega ) \delta a^{out} (\omega ') \rangle$. As we are seeking optimal values of squeezing, we should optimize the spectrum by solving $dS_{\theta }(\omega )/d\theta =0$. Then, the optimal value $\theta _{opt}$ satisfies $e^{2i\theta _{opt}}=-C_{aa}^{out}(\omega )/|C_{aa}^{out}(\omega )|$. Consequently, the optimized squeezing spectrum can be expressed as $S_{opt}(\omega )=1+2[C_{a^{\dagger }a}^{out}(\omega )-|C_{aa}^{out}(\omega )|]$. Using the input-output relation given above we get:

34 $$\begin{aligned} C_{aa}^{out}( \omega ) =&\kappa C_{aa}( \omega ) -\kappa M( \alpha _{1}( \omega ) +\alpha _{1}( -\omega ) ) -\kappa ( N \alpha _{4}( \omega ) \nonumber \\&+( N+1) \alpha _{4}( -\omega ) )+M, \end{aligned}$$

where $$ C_{aa}( \omega )$$ reads:

35 $$\begin{aligned} C_{aa}( \omega ) =&\kappa M \alpha _{1}( \omega ) \alpha _{1}( -\omega ) +\kappa ( N+1) \alpha _{1}( \omega ) \alpha _{4}( -\omega ) \nonumber \\&+\kappa N \alpha _{4}( \omega ) \alpha _{1}( -\omega ) +\kappa M \alpha _{4}( \omega ) \alpha _{4}( -\omega ) \nonumber \\&+\gamma \alpha _{2}( \omega ) \alpha _{5}( -\omega ) +\gamma _{m}(n_{th}+1) \alpha _{3}( \omega ) \alpha _{6}( -\omega ) \nonumber \\&+\gamma _{m} n_{th} \alpha _{6}( \omega ) \alpha _{3}( -\omega ). \end{aligned}$$

The covariance function, $C_{a^{\dagger }a}^{out}( \omega )$, is already determined in Sect. “Intensity spectrum and hybrid resonances”. Then, we obtain the spectrum for optimum output fields:

36 $$\begin{aligned} S_{opt}( \omega )=&1+2N -2| M+\kappa C_{aa}( \omega ) -\kappa M( \alpha _{1}( \omega ) \nonumber \\&+\alpha _{1}( -\omega ) ) -\kappa ( N \alpha _{4}( \omega ) +( N+1) \alpha _{4}( -\omega ) ) | \nonumber \\&+2\kappa C_{a^{\dagger }a}( \omega ) -4\kappa N{Re}(\alpha _{1}( -\omega ) ) \nonumber \\&-4\kappa M{Re}( \alpha _{4}(-\omega ) ). \end{aligned}$$

Experimentally, to measure the squeezing spectrum using a quantum-well cavity can be realized as follows. A high finesse microcavity with GaAs/AlAs samples is needed. The cavity contains an InGaAs quantum well possessing a low indium content^64,65^. Via a homodyne detection system, the produced light by the system is detected in the normal direction to the sample^66,67^. Then, emitted photons are mixed with a local oscillator on a beamsplitter. After that, beams outgoing the beamsplitter are focused on two photodetectors. By means of an RF spectrum analyzer, the frequency spectrum is analyzed^68^.

### Squeezing and frequency

**Figure 5:**
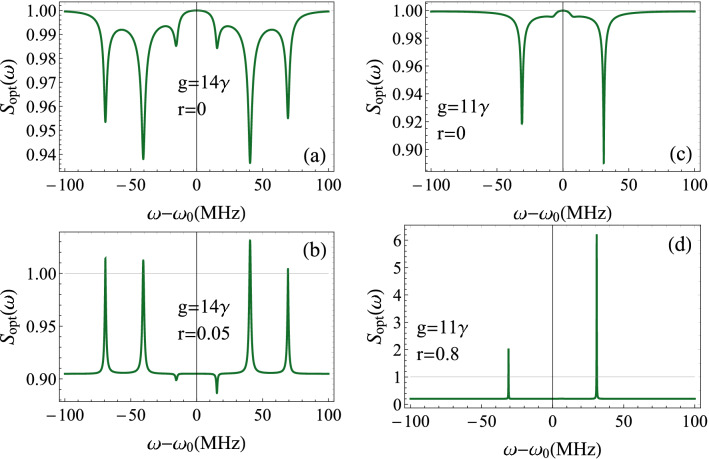
Noise spectrum of the transmitted radiation $S_{opt}(\omega )$ as a function of the frequency $\omega -\omega _{0}$ for $\gamma =3.6$ MHz, $\gamma _{m}=40$ Hz, $\kappa =2\pi \times 10^{5}$ Hz, $g_{0}=300$ Hz, $n_{th}\simeq 833$ corresponding to a reservoir temperature $T=0.4$ K, $\Delta =\omega _{m}=2\pi \times 2.7$ MHz, $\varepsilon =1.6\times 10^{8}\kappa$ and $\Delta _{b}=0$. We considered two values of photon-exciton coupling $g=14\gamma$ and $g=11\gamma$. (**a**), (**c**) The squeezing due to the optomechanical coupling. (**b**), (**d**) show the effect of the squeezed vacuum. The resonant peaks correspond to the squeezed radiation frequencies, but also to some fluctuations that could appear in the field due to the squeezed vacuum.

We choose first the situation where the system exhibits six peaks in its intensity spectrum as shown in Fig. 4a. In Fig. 5a, we plot $S_{opt}(\omega )$ as a function of $\omega -\omega _{0}$ for $g=14\gamma$ and $r=0$. The squeezing spectrum is formed of six distinct peaks corresponding to squeezed radiation. We observe weak squeezing at the optomechanical frequencies and a little stronger squeezing at the hybrid peaks reaching $6\%$. When the squeezed photons come into play, the situation profoundly changes (Fig.  5b). First, the coherent states are transformed to squeezed ones. Then, the hybrid peaks correspond now to minimal squeezing and are accompanied with small fluctuations. However, the optomechanical peaks present a little higher squeezing.

Secondly, we consider the case of hybrid resonance regime. This is obtained by decreasing the exciton-photon coupling to $g=11\gamma$ (Fig. 5c). The spectrum of noise shows two hybrid resonant peaks of squeezed light. Away from these frequencies, light is coherent identified by $S_{opt}=1$. It is noteworthy to mention here that the optimum squeezing is realized at the hybrid resonance frequencies despite that the optomechanical coupling is the origin of the nonlinearity in our system. When the squeezed vacuum acts on the cavity, the peaks are completely inverted and the squeezing is minimal at hybrid frequencies, with increasing fluctuations above the shot noise. Interestingly, the previous coherent states are transformed to strongly squeezed states in wide frequency windows with a $80\%$ of squeezing (Fig. 5d). This result seems very useful in the sense that we can achieve a very strong squeezed light, just we have to avoid the hybrid resonances and to tune to any other frequency.

### Squeezing and detuning

**Figure 6:**
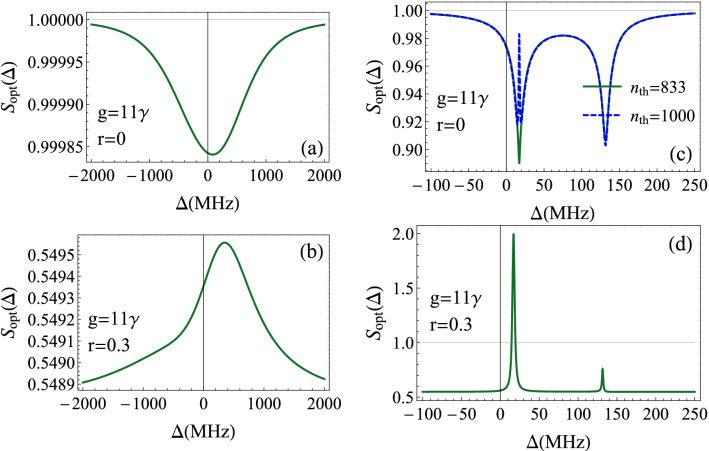
Noise spectrum $S_{opt}(\Delta )$ as a function of the detuning $\Delta$. We have choose two particular frequencies. (**a**), (**b**) $\omega -\omega _{0}=0$. (**c**), (**d**) $\omega -\omega _{0}=\Omega _{m}$ corresponding to the hybrid resonance regime. The other parameters are the same as in Fig. 4.

Here, we investigate the effect of the detuning on the squeezed radiation. For this purpose, we focus on two particular situations. The first corresponds to zero frequency $\omega - \omega _{0}=0$, while the second is for $\omega -\omega _{0}\approx 31.02$MHz =$\Omega _{m}$ giving maximal (minimal) squeezing in Fig. 5c, d, which corresponds to the hybrid resonance regime.

When $\omega -\omega _{0}=0$, we observe very small variation of the squeezing, where the maximum is produced for a quasi-resonant excitation (Fig. 6a). When applying the squeezed vacuum, the whole spectrum is inverted and translated down, and we attain stronger levels of squeezed light, approaching $45\%$ in the chosen range of the system parameters (Fig. 6b).

When the system reaches the hybrid resonance regime, $\omega -\omega _{0}= \Omega _{m}$, the spectrum is illustrated by Fig.  6c. As a first ascertainment, we observe two peaks of squeezing where the maximum corresponds to the frequency of the cooling regime close to the quantum ground state $\Delta =\omega _{m}$, whereas the other to $\Delta \approx 130$MHz (solid line). For higher thermal bath temperature, $n_{th}=1000$, the squeezing at $\Delta =\omega _{m}$ is reduced and is no longer maximal at this frequency. However, the other peak is still unaffected and exhibits a stronger resistance against the high thermal excitations (dashed line). Then, by injecting the squeezed photons inside the cavity, the frequency detunings $\Delta =\omega _{m}$ corresponds now to minimal squeezed radiation, and some fluctuations appear. The other peak is still under the shot noise level. Here, we also confirm the passage of the coherent states of light to highly squeezed states (Fig. 6d).

Compared to the traditional quantum well cavity (polariton cavity), the comportment of the present scheme is quite different. Indeed, in the absence of any external squeezed source, and due to excitonic nonlinearity, the best squeezing of the polariton cavity is obtained at zero frequency, while in our system the squeezing is minimal at zero frequency.

### Squeezing and squeeze parameter

**Figure 7:**
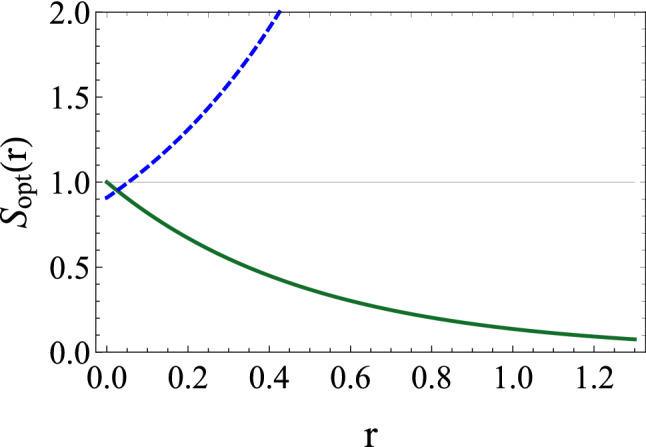
Noise spectrum $S_{opt}(r)$ as a function of the squeeze parameter *r* for $g=11\gamma$. Solid line for $\omega -\omega _{0}=0$, and dashed line for $\omega -\omega _{0}=\Omega _{m}$. The other parameters are the same as in Fig. 4.

To gain deep insight of the study, we need to see the effect of the squeeze parameter *r* more closer. In Fig. 7, we represent $S_{opt}$ as a function of *r* by considering the two particular frequencies discussed in the previous section. It is clearly shown that at the hybrid resonant frequencies, the transmitted radiation exhibits strong fluctuations (dashed line). However, at zero frequency the situation is always favorable to stronger squeezing as the squeeze parameter increases (solid line). Moreover, a perfect squeezing is predicted with sufficiently strong injection of squeezed photons.

### Squeezing and optomechanical coupling

**Figure 8:**
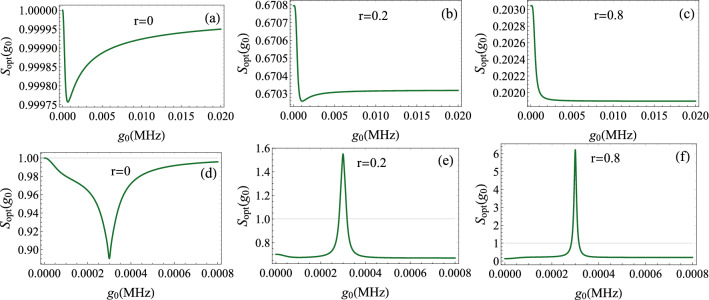
Noise spectrum $S_{opt}(g_{0})$ as a function of the optomechanical coupling $g_{0}$ for $g=11\gamma$. (**a**), (**b**) and (**c**) $\omega -\omega _{0}=0$. (**d**), (**e**) and (**f**) $\omega -\omega _{0}=\Omega _{m}$. The other parameters are the same as in Fig. 4. In the hybrid resonance regime (Figs. (d), (e) and (f)), the spectrum is inverted and the squeezing peak is narrowed when increasing the squeeze parameter *r*.

Now, we focus on the effect of the optomechanical coupling as it is the primal source of squeezing in this system. We conclude from the illustration of Fig. 8a that at zero frequency and for the chosen parameters, the squeezing varies very weakly as a function of $g_{0}$. The effect of the nonlinear optomechanical interaction is minor. By coupling the cavity to the squeezed reservoir, a much higher amount of squeezing is attainable (Fig. 8b, c). In this case, the effect of the squeezed vacuum dominates that of the nonlinear optomechanical coupling.

**Figure 9:**
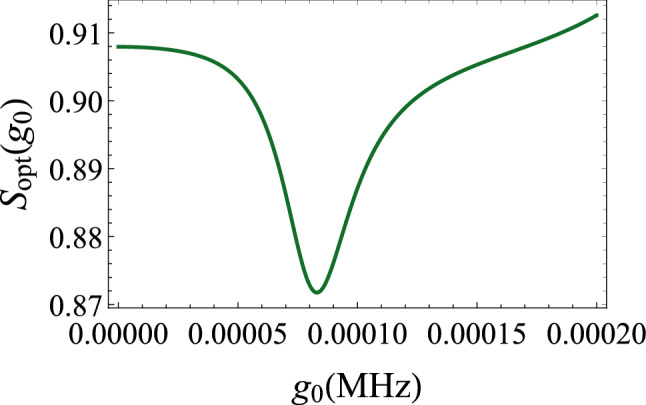
Optimum noise spectrum $S_{opt}(g_{0})$ versus the optomechanical coupling $g_{0}$ for $r=0.05$. The other parameters are the same as in Fig. 4.

At the hybrid frequency $\Omega _{m}$, we observe first that for $r=0$ the squeezing is more than $10\%$ (Fig. 8d). By increasing *r* to 0.2, the squeezing may reach over than $30\%$. This elevation is accompanied with some fluctuations appearing in a single peak form (Fig. 8e). For stronger squeeze parameter, the squeezing may approach $80\%$, at the expense of increasing fluctuations above the shot noise level (Fig. 8f). We also notice a narrowing of the peaks each time that *r* increases.

It is important to mention here that except a certain range of $g_{0}$ giving high fluctuations, we can achieve a strong and stable squeezing at any given value of optomechanical coupling.

The squeezed vacuum (external source) is used to improve the efficiency of the squeezing generated by the optomechanical coupling . For example, we plot in Fig. 9 the noise spectrum $S_{opt}$ as a function of the optomechanical coupling $g_{0}$ for a fixed value of the squeeze parameter $r=0.05$. We observe that when $g_{0}=0$, the squeezing due to the squeezed vacuum is about $9.2\%$. As $g_{0}$ increases, we can achieve higher squeezing that may reach $13\%$. In this situation, the squeezing of the output field is enhanced compared to the input of the squeezed reservoir.

## Conclusion

We have studied the photon correlations and the noise properties of the transmitted radiation by a hybrid system composed of an optomechanical resonator and an optical cavity including a quantum well, where the cavity interacts with a squeezed vacuum. We have shown that the presence of excitons destroys the optical bistability. The application of the squeezed vacuum modifies the transmitted intensity spectrum and affects the characteristic frequencies of the system. Away from the hybrid resonance regime, the squeezing spectrum consists of six peaks involving mechanical, excitonic and cavity modes. When the system attains the hybrid regime, the spectrum shows two hybrid resonant peaks corresponding to maximal squeezing. Interestingly, the coupling to the squeezed vacuum strongly enhances and stabilizes the degree of squeezing in the outgoing light. However, to obtain the maximal squeezing, we should avoid the characteristic resonant frequencies of the system which do not correspond anymore to the maximal squeezing, but to high fluctuations, in the presence of the squeezed vacuum.
